# Iron Deficiency Prevention and Dietary Habits Among Elite Female University Athletes in Japan

**DOI:** 10.3390/sports13070220

**Published:** 2025-07-07

**Authors:** Hiromi Inaba, Haruo Hanawa, Fumi Hoshino, Mutsuaki Edama, Go Omori

**Affiliations:** 1 Athlete Support Research Center, Niigata University of Health and Welfare, 1398 Shimami-cho, Kita-ku, Niigata 950-3198, Japan; 2 Department of Health and Nutrition, Faculty of Health Science, Niigata University of Health and Welfare, Niigata 950-3198, Japan; 3 Department of Clinical Engineering and Medical Technology, Niigata University of Health and Welfare, Niigata 950-3198, Japan; 4 Institute for Human Movement and Medical Sciences, Niigata University of Health and Welfare, Niigata 950-3198, Japan; 5 Department of Health and Sports, Niigata University of Health and Welfare, Niigata 950-3198, Japan

**Keywords:** anemia, female athlete, eating habits, hemoglobin

## Abstract

This study investigated the percentage of iron deficiency anemia (IDA) and iron deficiency (ID) among 71 elite female athletes at a Japanese university and assessed their dietary habits. IDA was identified in 9.9% (*n* = 7) of participants, and only 22.5% (*n* = 16) self-reported dietary practices aimed at preventing or managing ID/IDA. Notably, 52.1% (*n* = 37) of the athletes exhibited IDA or ID but lacked an appropriate dietary approach. Moreover, even among those who reported an intentional dietary approach to the prevention or management of ID/IDA, the intake of iron- and vitamin C-rich foods was insufficient, limiting the effectiveness of their efforts. These findings highlight a gap between awareness and effective practice, indicating that many female athletes in Japan, despite being at elevated risk, do not follow evidence-based dietary strategies for preventing or treating ID/IDA. Targeted nutritional education and routine screening of iron status are strongly recommended for this population.

## 1. Introduction

Iron is a component of all living cells and is recognized as an essential element for maintaining health. It is involved in various biochemical reactions, including oxygen transport (as a component of hemoglobin), ATP production, DNA synthesis, and electron transfer [1,2]. Most of the body’s iron (approximately 65%) is found in hemoglobin, which is present in red blood cells. Other locations include myoglobin in the muscles, enzymes, cytochromes, reticuloendothelial system (RES) macrophages, and bone marrow. The stored portion is present in liver cells as ferritin [3,4]. Iron metabolism is among the most intricate processes within the human body, encompassing numerous organs and tissues, including the intestines, bone marrow, spleen, and liver [5]. Iron is efficiently stored through the body’s homeostasis mechanisms; however, iron deficiency may occur when intake falls below physiological requirements or stores are depleted. Individuals considered to be at high risk for iron deficiency include premenopausal women; infants; elderly hospitalized patients who undergo frequent blood draws for diagnostic purposes; individuals with gastrointestinal bleeding, malabsorption states, and/or gastric cancer; and those who have undergone gastrointestinal surgery [6].

Iron deficiency anemia (IDA) and iron deficiency (ID) are the most common micronutrient disorders worldwide [7,8]. Iron is involved in multiple cellular functions and physiological systems, making it essential for human health. ID is a leading contributor to the global burden of disease, particularly affecting children, premenopausal women, and people living in low- and middle-income countries [9,10]. Additionally, IDA and ID are common nutrient deficiencies among athletes, especially in endurance-trained athletes [11], and ID is known to significantly contribute to reduced performance [11,12,13]. The iron requirements of athletes may be higher due to the increased erythropoietic drive caused by regular exercise. Moreover, increased iron losses (i.e., through the gastrointestinal tract, hematuria, and sweat) [14], poor dietary iron intake [15], foot strike hemolysis [16], exercise-induced inflammation [17], and environmental factors such as hypoxia may all influence iron metabolism in athletes [18]. In particular, female athletes are at risk for ID due to menstruation; thus, ID screening is widely recommended for all athletes [19,20,21]. In fact, women lose approximately 1 mg of iron per day during menstruation, which may be higher for heavy menstrual bleeding, where blood loss is estimated to be 5–6 times greater [22]. However, despite the importance of iron, ID remains highly prevalent, globally ranging between 9% and 60% in female athletes [23,24].

Low Hb concentrations can result in reduced oxygen (O_2_) transport to working muscles; this is the primary mechanism for reduced performance due to anemia. More specifically, these reduced performance outcomes include decreases in maximal O_2_ consumption (VO2max) and aerobic power [19]. IDA can also significantly impact performance depending on its severity, whereas the impact of non-anemic iron deficiency (NAID; Hb ≧ 12 g/dL and Ft < 30 μg/L) is less clear, although some reports have investigated it [25]. One study evaluated the relationship between serum Ft and 2 km time trial performance among female collegiate rowers at the beginning of a competition season, with athletes with NAID having slower trial times compared to those with sufficient iron stores [26]. Moreover, several studies have demonstrated increased aerobic capacity indices after iron supplementation in female athletes [27,28], which implies that performance was affected by NAID.

Oral iron supplementation is the first-line treatment for IDA and ID, and adequate iron intake is also paramount to their prevention [29]. Iron is available in many supplemental forms to support the higher recommended dietary iron intake of female athletes. According to the Dietary Reference Intakes for Japanese (2020), the recommended dietary allowance for iron is 10.5 mg/day for women aged 18–49 years with menstruation [30]. In Japan, menstrual iron loss is estimated to be approximately 0.55 mg day, corresponding to approximately 15.5 mg per menstrual cycle. Premenopausal adult women need to account for the additional iron loss from menstruation [31]; thus, the reference nutrient intake for adult females in the UK is 14.8 mg/day [32], while the recommended dietary allowance in the US is set at 18 mg/day [33].

A strategic approach to iron ingestion (e.g., the timing of iron intake) could enhance iron absorption in this population, which has become a topic of interest for examining ID in athletes [34]. Athletes with ID are commonly recommended to take 60–120 mg/day of elemental iron given for 2 months, varying based on ID severity and individual tolerance from a gastrointestinal perspective [35,36]. In Japan, 100–200 mg/day is commonly prescribed for ID and IDA treatment [37]. However, some athletes stop taking medications due to significant gastrointestinal side effects [38].

Improvements in hematological biomarkers have been documented in response to iron supplementation in athletes with ID [23,39]. However, the effectiveness of iron supplementation on physical parameters beyond endurance performance and maximal aerobic capacity is unclear, which is particularly important in sports requiring substantial strength and power (e.g., weightlifting, American football). Thus, aside from iron supplementation, consuming foods with high iron content (e.g., liver, eggs, red meat, fish, tofu, and spinach) is also important for preventing ID and IDA. It is also important to consume vitamin C, which promotes iron absorption. Moreover, avoiding iron absorption inhibitors, including tannins, calcium, oxalates, and phytic acid, can help prevent the reduction in iron bioavailability [40]. Although there are few studies on athletes with specific diets to prevent ID, further studies in this field are needed. In addition, no studies have examined the actual eating awareness and behavior of athletes with dietary habits specifically aimed at preventing IDA/ID.

This study aimed to investigate IDA/ID percentages among female athletes in one elite Japanese university and clarify the dietary awareness, eating habits, and iron nutritional status of athletes who self-report having specific dietary habits at IDA/ID prevention.

## 2. Materials and Methods

### 2.1. Participants and the Survey Period

A total of 71 female university athletes from competitive sports clubs (e.g., basketball, volleyball, swimming, soccer, and track and field) participated in this cross-sectional study between December 2022 and February 2023. Participants were recruited for the survey by the co-author in collaboration with team trainers. All 71 individuals provided informed consent before participation, resulting in a 100% response rate. Participants unable to undergo physical measurements on the day of the survey due to injury were eligible for exclusion; however, no participant met these exclusion criteria. The surveys were conducted during the training period. All teams competed at the national level in Japan (classified as Tier 3 competition) [41]. The participants were informed about the purpose, procedures, and potential risks of the study, and written informed consent was obtained in advance. Participation was voluntary, with the option to withdraw at any time.

### 2.2. Ethical Considerations

This study was conducted per the Declaration of Helsinki [42] and the Ethical Guidelines for Medical Research Involving Human Subjects (Ministry of Education, Culture, Sports, Science and Technology and Ministry of Health, Labor and Welfare 2017) [43]. The study protocol was reviewed and approved by the Institutional Review Board of Niigata University of Health and Welfare (approval number: 18831-220526).

### 2.3. Anthropometry

Height (cm), body weight (kg), and body fat (%) were measured using a digital height meter (AD6400, A&D, Tokyo, Japan) or bioelectrical impedance method (InBody470, Inbody Japan, Tokyo, Japan). The body mass index (BMI) was calculated as body weight (kg)/height (m^2^).

### 2.4. Methods and the Dietary Survey

Two questionnaires assessed participants’ dietary habits. First, a 12-item survey examined the weekly frequency of food intake behaviors (maximum 7 days/week). Items included meal consumption frequency (e.g., breakfast), staple foods, major food groups (e.g., vegetables, meat, dairy), and snack intake [44].

The Brief Self-Administered Diet History Questionnaire (BDHQ) [45,46] was used to estimate energy and nutrient intake. This self-report tool assesses the intake frequency of 58 food and beverage items over the previous month. Participants respond using a seven-point scale to indicate how often they consumed each item [47]. Kobayashi et al. reported that the BDHQ has acceptable validity for ranking nutrient and food group intake among Japanese adults [45]. In particular, they found a Pearson correlation coefficient of 0.62 between iron intake estimated by the BDHQ and that from 16-day dietary records, supporting the BDHQ’s reliability in assessing iron intake. Participants used food models of typical Japanese rice bowl sizes (150–350 g) and a rice ball (100 g) to improve the accuracy of rice intake estimation. This helped address common under- or overestimation, especially among athletes. In the BDHQ, one serving of rice is assumed to be 150 g. The completed questionnaires were returned by mail to Gender Medical Research Inc., where dietary data were analyzed using a standardized scoring algorithm.

Two additional items were assessed via a questionnaire. First, participants were asked whether they usually followed a diet mindful of preventing or managing IDA or ID, with responses categorized as “yes” or “no.” Second, information regarding the product name, intake timing, and frequency of use was collected and added to the BDHQ result for those who reported dietary supplement use. Energy and nutrient intake were calculated per kg of body weight.

Staff, such as coaches and trainers, were not present to ensure unbiased responses. Two registered dietitians were available to assist participants with the BDHQ and rice portion estimation.

### 2.5. Biochemical Tests

Blood samples were collected via venipuncture in the morning, and each item was measured within 3 h after collection. Dietary and physical activity restrictions were not imposed. Red blood cell count (RBC), Hb, and hematocrit (Ht) were measured using a multi-parameter automated blood cell analyzer XR-1000 (Sysmex Corporation, Hyogo, Japan) with whole blood placed in an EDTA blood collection tube. Serum iron, unsaturated iron-binding capacity (UIBC), and serum Ft were measured using serum. The measurement equipment used was an automated analyzer LABOSPECT006 (Hitachi High-Tech Corporation, Tokyo, Japan).

### 2.6. Proportion of Subjects with Anemia and Iron Deficiency Stages

Subjects were classified based on the staging classification of the Swiss Society of Sports Medicine for anemia/ID as follows: normal (Hb ≧ 12 g/dL and Ft ≧ 30 μg/L), NAID (Hb ≧ 12 g/dL and Ft < 30 μg/L), ID with microcytosis and hypochromia (IDMH, Hb ≥ 12 g/dL, Ft < 30 μg/L, MCH < 28 pg, MCV < 80 fL), IDA (Hb < 12 g/dL and Ft < 30 μg/L), and non-iron deficiency anemia (NIDA, Hb < 12 g/dL and Ft ≧ 30 μg/L) [35].

### 2.7. Statistical Analysis

Data were analyzed using R (version 4.1.0.). Statistical analyses were conducted to compare two groups based on responses to the following question: “Do you consume a diet mindful of preventing or treating IDA daily?” An independent t-test was conducted to analyze differences in physical measurements, blood test results, and quantitative and qualitative food frequency questionnaire responses. Fisher’s exact test with Holm correction was applied to compare anemia prevalence according to dietary habits. The significance level was set at *p* < 0.05.

## 3. Results

Table 1 presents the physical characteristics of the participants. The mean age was 20.2 ± 1.0 years (mean ± SD). Out of 71 participants, 16 (22.5%) had dietary habits aimed at preventing or treating ID, while 55 (77.5%) did not, and these groups had no differences in height, body mass, BMI, and body fat percentage. Regarding their sports clubs, the highest number of participants were part of the basketball club (*n* = 21), followed by volleyball (*n* = 16), swimming (*n* = 13), soccer (*n* = 12), and track and field (*n* = 9) (Table 2). Among athletes who followed dietary practices to prevent ID, the highest and lowest percentages were seen among soccer players (58.3%) and swimmers (7.7%), respectively.

IDA was identified in 9.9% (*n* = 7) of the surveyed female college athletes. The proportion of athletes with normal iron nutritional status was the highest among basketball players (35.0%) and the lowest among volleyball players (17.6%) (Table 3). Table 4 presents the percentage of anemia and ID stages according to the presence of dietary habits for preventing or treating anemia. The IDA rate was 18.8% (3/16) and 7.3% (4/55) in the athletes with dietary habits for preventing or treating anemia and those who did not follow a diet, respectively.

Table 5 presents the hematologic data and iron status of the female athletes grouped based on their dietary habits. Both Hb and Ht were significantly lower in the group with dietary practices to prevent ID than those who did not follow a diet (Hb: 12.6 ± 0.9 vs. 13.1 ± 0.9 g/dL, *p* < 0.034; Ht: 38.1% ± 2.2% vs. 39.8% ± 2.6%, *p* < 0.028). The remaining anemia-related parameters, such as MCV, MCH, MCHC, RBC, serum iron, and serum Ft, were similar between the two groups.

The qualitative food frequency survey results are shown in Table 6. Athletes who actively engaged in IDA prevention had a significantly greater frequency of abstaining from sweets and soft drinks compared to those who were not (4.13 ± 2.34 vs. 2.73 ± 2.18 days per week, *p* = 0.049). The intake frequency of light-colored vegetables (e.g., cucumbers, cabbage, lettuce, etc.) was also significantly greater among athletes with dietary practices versus those without (5.34 ± 1.05 vs. 4.67 ± 1.54 days per week, *p* = 0.048). There were no significant differences between the two groups in the frequency of meat, fish, and egg intake and the frequency with which breakfast was eaten.

The two groups had no significant differences in energy and iron intake, as well as other nutrients such as carbohydrates, protein, and vitamin C (Table 7). The daily iron and vitamin C intake was 6.1 ± 2.5 and 77.3 ± 50.5 mg/day, respectively. Two participants used supplements to increase their iron intake.

## 4. Discussion

In this study, 22.5% (*n* = 16) of the athletes self-reported regularly following dietary practices to prevent or treat IDA. However, these athletes actually had significantly lower Hb and Ht than those not on a watchful diet. This may be due to athletes previously diagnosed with IDA/ID adhering to iron-conscious dietary practices. Additionally, 52.1% (*n* = 37) of athletes with ID/IDA did not have dietary practices that addressed ID/IDA. Notably, the athletes with dietary practices for ID/IDA ate light-colored vegetables and abstained from sweets more frequently. No significant differences were found in nutrient intake, including iron and vitamin C, or energy between the two groups.

Our survey of elite female college athletes in Japan uncovered a 9.9% IDA, while only 29.6% were normal. Several previous studies have also evaluated Japanese female athletes. In 2011, Komatsu et al. investigated IDA among athletes competing in the Universiade, reporting a 1.7% prevalence that decreased yearly, although the analysis was only based on Hb [48]. Meanwhile, another study found that 3.4% of elite Japanese high school long-distance female runners had IDA [49]. In other countries, Koehler et al. reported that 6.2% of female athletes (16.2 ± 2.7 years old) in Germany had Hb levels below 12 g/dL [50], while 5.7% of female athletes enrolled in National Collegiate Athletic Association Division I institutions in the United States had IDA [51]. Lastly, Ponorac et al. reported that 3.5% of professional athletes (*n* = 85) in handball, volleyball, soccer, and judo competing at the highest level in Bosnia and Herzegovina had IDA [52]. Compared to these previous studies, the IDA percentage in our study cohort is relatively high. Consequently, elite female university athletes would also benefit from the regular screening of their iron status and levels.

There are various causes of IDA, with low dietary iron intake (i.e., less than 6.1 ± 2.5 mg/day) considered one of the main causes. Treatment can significantly improve a patient’s quality of life even in mild IDA cases, and dietary habits that aim to prevent or treat IDA are very important [53]. ID treatments include oral supplements, intravenous injections, and dietary iron treatments such as diet modification, dietary advice and counseling, and incorporating iron-fortified or naturally iron-rich products into the daily diet [54]. Dietary modification is the preferred strategy for ensuring adequate iron intake and maintaining iron status and is the first line of action in iron deficiency prevention in female athletes [55,56]. Some examples of high-iron foods regularly consumed in the Japanese diet include meat, fish, eggs, soybean products (e.g., tofu, natto, miso, etc.), and komatsuna. However, these were not frequently consumed by the participants in this study (Table 6). This highlights the need for nutritional education among athletes on the importance of intake timing and food quantity and quality for performance optimization. The dietary habits of our participants were generally unsuitable for IDA or ID treatment or prevention; thus, further nutritional guidance is needed.

There are two forms of dietary iron: heme iron, sourced from Hb and myoglobin in animal-based food, and non-heme iron, present in both plant and animal tissues. Heme iron has greater bioavailability than non-heme iron (15–35% vs. 2–20%) [57,58] because it is more efficiently absorbed by specific, high-affinity, mucosal brush-border heme-binding iron sites [57,58]. Since non-heme iron characteristically comprises most of the total dietary iron intake, its low bioavailability may pose a problem for athletes [34]. Notably, both phenolic compounds (e.g., polyphenols and tannins contained in tea, coffee, and other plant foods) and phytates (e.g., whole-grain cereals, legumes, and nuts) bind with non-heme iron and dose-dependently inhibit its absorption [59]. This study did not investigate the intake of green tea, black tea, or coffee, which have high polyphenol contents, in detail, which is a topic for future investigation.

Ascorbic acid may help overcome some of the inhibitory effects of confounding nutrients on non-heme iron absorption, with 50 mg of ascorbic acid shown to increase non-heme iron absorption up to threefold [60]. However, the participants in this study had a relatively low vitamin C intake (77.3 ± 50.5 mg/day) compared to the recommended amount of 100 mg in the Japanese Dietary Reference Intakes 2020. Japanese college athletes have limited time for part-time work due to their long practice hours. Therefore, it is recommended that registered dietitians encourage athletes to increase their vitamin C intake by consuming vitamin C-rich foods and beverages, such as fresh fruit, 100% orange juice, grapefruit juice, and acerola-based drinks. The continued provision of such dietary guidance by registered dietitians may help enhance vitamin C intake among athletes.

The dietary habits of the participating athletes exhibited three notable characteristics. First, the snack consumption frequency recommended for athletes [20] was low, averaging approximately two days per week. Second, some athletes did not adhere to the basic dietary principle of consuming three meals per day. Third, meat and fish intake, key dietary sources of both protein and iron, was low among the participants. Also, even athletes with dietary habits for IDA prevention had a very low weekly intake of green and yellow vegetables and fruits (3.70 ± 1.72 and 3.56 ± 2.00 days per week, respectively, as shown in Table 6). Since many dietary factors and homeostatic regulators influence dietary iron bioavailability, it is reasonable to expect daily and interindividual variance in the dietary iron requirements of athletes, making it hard to implement a one-size-fits-all approach [34]. Nevertheless, the increased dietary intakes of both iron and vitamin C are the most effective treatments for IDA. Participants in this study had low intakes of foods high in iron and vitamin C, such as fruits and green and yellow vegetables. Interestingly, although these participants claimed to be conscious of preventing and treating IDA/ID, they did not proactively consume iron-rich foods. Instead, they avoided sweets and consumed more light-colored vegetables than the other group (Table 6). This eating habit is similar to the eating habits of people in Japan who are trying to lose weight. Evidently, the dietary practices of these athletes were not actually suitable for IDA/ID prevention. Thus, appropriate nutritional education from a registered dietitian with expert knowledge is necessary. This conclusion is based on the observed low intake frequency of iron-rich foods and vitamin C-rich fruits and vegetables and the absence of proactive dietary strategies to enhance iron absorption.

This study had limitations. It was conducted in a single university in a rural city of Japan; therefore, we were unable to adequately consider the area of residence, the season of the study period, and intraindividual variation. Additionally, all self-reported dietary survey methods are subject to reporting errors, especially for athletes. Therefore, it is important to keep in mind that the actual intake of nutrients and energy may be slightly higher. We did not investigate whether the participants had received nutritional guidance from a registered dietitian in the past regarding IDA prevention or treatment; for athletes who may have had received previous nutritional guidance, it was necessary to ascertain what kind of nutritional guidance they received (for example, whether it was for weight loss, related to the female athlete triad, or related to hydration). Participant selection criteria were not sufficiently addressed in this study. Ideally, factors specifically associated with IDA should have been evaluated. In particular, vegetarians, people with religious dietary restrictions, and those actively trying to lose weight should have been identified through screening questions and excluded as necessary. The use of oral contraceptives and nonsteroidal anti-inflammatory drugs (NSAIDs) should also have been taken into consideration. In addition, participants were required to restrict their diet and physical activity before blood collection. Ferritin levels may have been elevated if participants engaged in strenuous physical activity before blood collection, which could have influenced the results. Based on the findings of this study, future research will aim to develop and refine nutritional support strategies tailored to the needs of athletes.

## 5. Conclusions

Iron deficiency anemia (IDA) was present in 9.9% (*n* = 7) of the surveyed female college athletes. Only 22.5% (*n* = 16) reported dietary habits for preventing or treating ID/IDA, while 52.1% (*n* = 37) of those diagnosed with IDA or ID lacked such dietary practices. Furthermore, even among those with intentional dietary habits, the intake of iron- and vitamin C-rich foods was insufficient, reducing the effectiveness of their efforts. These results reveal a significant gap between awareness and the effective implementation of dietary strategies. Given the high risk of ID/IDA in this population, targeted nutritional education and routine iron status screening are strongly recommended.

## Figures and Tables

**Table 1:** Physical characteristics of female athletes grouped based on dietary habits.

	Dietary Practices to Prevent or Treat Iron Deficiency		
	No	Yes	*p*-Value
	*n* = 55, 77.5%	*n* = 16, 22.5%	
Age, year	20.2 ± 1.0	20.2 ± 1.1	0.939
Height, cm	164.8 ± 5.6	162.8 ± 5.8	0.227
Body Mass, kg	59.1 ± 6.8	57.6 ± 5.9	0.227
BMI, kg/m^2^	21.8 ± 1.9	21.7 ± 1.0	0.893
Body Fat, %	22.8 ± 4.7	22.8 ± 3.8	0.994

Data are presented as the mean ± SD. BMI, body mass index.

**Table 2:** Sports clubs of female athletes grouped based on dietary habits.

	Use of Self-Reported Dietary Practices to Prevent or Treat Anemia			
Club	No		Yes	
	*n* = 55, 77.5%		*n* = 16, 22.5%	
Basketball	17	(30.9%)	4	(25.0%)
Soccer	5	(9.1%)	7	(43.8%)
Volleyball	14	(25.5%)	2	(12.5%)
Track and field	7	(12.7%)	2	(12.5%)
Swimming	12	(21.8%)	1	(6.3%)

**Table 3:** Anemia and stages of iron deficiency among female athletes grouped based on sports clubs.

Club	Normal		NAID		IDMH		IDA		NIDA	
Basketball	7	(35.0%)	11	(55.0%)	0	(0.0%)	2	(10.0%)	0	(0.0%)
Soccer	5	(41.7%)	5	(41.7%)	0	(0.0%)	1	(8.3%)	1	(8.3%)
Volleyball	3	(17.6%)	10	(58.8%)	0	(0.0%)	4	(23.5%)	0	(0.0%)
Track and field	2	(22.2%)	7	(77.8%)	0	(0.0%)	0	(0.0%)	0	(0.0%)
Swimming	4	(30.8%)	9	(69.2%)	0	(0.0%)	0	(0.0%)	0	(0.0%)

NAID: non-anemic iron deficiency; IDMH: iron deficiency with microcytosis and hypochromia; IDA: iron deficiency anemia; NIDA: non-iron deficiency anemia without reduced serum ferritin.

**Table 4:** Iron deficiency stages among female athletes grouped based on dietary habits.

Diagnosis	Use of Self-Reported Dietary Practices to Prevent or Treat Anemia				
	No		Yes		*p*-Value
	*n* = 55, 77.5%		*n* = 16, 22.5%		
Normal	18	(32.7%)	3	(18.8%)	1.000
NAID	33	(60.0%)	9	(56.3%)	
IDMH	0	(0.0%)	0	(0.0%)	
IDA	4	(7.3%)	3	(18.8%)	
NIDA	0	(0.0%)	1	(6.3%)	

Fisher’s exact test (Holm-adjusted *p*-values). NAID: non-anemic iron deficiency, IDMH: iron deficiency with microcytosis and hypochromia, IDA: iron deficiency anemia.

**Table 5:** Hematologic data and iron status of female athletes grouped based on dietary habits.

Items		Dietary Practices to Prevent or Treat Iron Deficiency		
		No	Yes	*p*-Value
		*n* = 55, 77.5%	*n* = 16, 22.5%	
Hb	g/dL	13.1 ± 0.92	12.6 ± 0.89	0.034
Ht	%	39.7 ± 2.6	38.1 ± 2.32	0.028
MCV	fl	91.2 ± 3.53	90.2 ± 2.9	0.275
MCH	pg	30.2 ± 1.18	29.8 ± 1.46	0.316
MCHC	%	33.1 ± 0.66	33 ± 0.8	0.562
Serum iron	μg/dL	97.0 ± 32.2	77.4 ± 34.7	0.062
RBC	×10^4^/μL	436 ± 33	422 ± 32	0.176
UIBC	μg/dL	250 ± 53	270 ± 61	0.250
Serum Ft	ng/mL	25.6 ± 17.2	24.0 ± 20.4	0.778

Data are presented as the mean ± SD. Normal: Hb ≧ 12 g/dL and Ft ≧ 30 μg/L.

**Table 6:** Qualitative food intake frequency survey results of female athletes grouped based on dietary habits.

	Dietary Practices to Prevent or Treat Iron Deficiency		
Number of Days Per Week	No	Yes	*p*-Value
	*n* = 55, 77.5%	*n* = 16, 22.5%	
Avoiding sweets and soft drinks (juice, etc.)	2.73 ± 2.18	4.13 ± 2.34	0.049
Eating 3 meals a day (breakfast, lunch, and dinner)	5.16 ± 1.99	5.88 ± 1.36	0.118
Eating staple foods (rice, bread, noodles, etc.)	6.36 ± 1.12	6.38 ± 1.17	0.973
Eating eggs (or egg dishes)	4.65 ± 2.05	4.94 ± 1.95	0.627
Eating meat and fish side dishes	5.04 ± 1.89	4.31 ± 2.17	0.249
Eating soy products such as tofu and natto	4.16 ± 1.95	4.44 ± 1.69	0.597
Eating vegetables high in beta-carotene (carrots, spinach, komatsuna, etc.)	3.73 ± 1.91	3.69 ± 1.72	0.939
Eating light-colored vegetables (cucumbers, cabbage, lettuce, etc.)	4.67 ± 1.54	5.38 ± 1.05	0.048
Eating milk/yogurt	3.87 ± 2.46	3.93 ± 1.64	0.905
Eating fruit (including 100% juice)	2.98 ± 2.01	3.56 ± 2.00	0.330
Eating breakfast	5.11 ± 2.09	5.69 ± 1.57	0.252
Eating snacks	1.95 ± 2.24	2.44 ± 2.44	0.475
Total	50.3 ± 13.1	54.8 ± 10.2	0.171

Participants answered in terms of the number of days per week (lowest: 0 days; highest: 7 days). Data are presented as the mean ± SD.

**Table 7:** Energy and nutrient intakes of female athletes grouped based on dietary habits.

Energy or Nutrients		Dietary Practices to Prevent or Treat Iron Deficiency		*p*-Value
		No	Yes	
		*n* = 55, 77.5%	*n* = 16, 22.5%	
Energy	kcal/BW kg	29.3 ± 9.81	27.2 ± 9.34	0.508
Protein	g/kg BW	0.97 ± 0.33	0.91 ± 0.29	0.598
Fat	g/kg BW	0.77 ± 0.28	0.70 ± 0.24	0.481
Carbohydrate	g/kg BW	4.42 ± 1.71	4.21 ± 1.60	0.669
Retinol equivalent	μgRAE/kg BW	7.20 ± 4.30	10.0 ± 11.7	0.392
Vitamin D	μg/kg BW	0.13 ± 0.09	0.11 ± 0.10	0.526
α-Tocopherol	μg/kg BW	93.3 ± 38.0	91.9 ± 38.0	0.914
Vitamin K	mg/kg BW	5.11 ± 2.65	5.08 ± 3.8	0.967
Vitamin B_1_	μg/kg BW	10.9 ± 4.2	11.3 ± 3.8	0.857
Vitamin B_2_	μg/kg BW	16.7 ± 6.5	15.6 ± 6.8	0.623
Vitamin B_6_	μg/kg BW	15.6 ± 6.6	16.3 ± 6.7	0.799
Vitamin B_12_	μg/kg BW	8.78 ± 5.81	8.31 ± 7.1	0.784
Folic acid	μg/kg BW	4.09 ± 1.97	4.72 ± 3.1	0.457
Pantothenic acid	μg/kg BW	96.9 ± 34.5	96.0 ± 32.7	0.580
Vitamin C	mg/kg BW	1.30 ± 0.78	1.52 ± 0.94	0.449
Na	mg/kg BW	56.6 ± 14.6	55.5 ± 19.4	0.833
Ca	mg/kg BW	6.42 ± 3.02	6.42 ± 3.06	0.993
Fe	μg/kg BW	104 ± 42	105 ± 5	0.849
Mg	mg/kg BW	3.19 ± 1.2	3.15 ± 1.26	0.913
K	mg/kg BW	30.4 ± 13.4	31.6 ± 14.9	0.805
P	mg/kg BW	13.9 ± 5.05	13.4 ± 4.3	0.678

The numbers indicate the amount of energy or nutrients per kilogram of body weight (BW). Data are presented as the mean ± SD.

## Data Availability

The data supporting the findings of this study are available from the corresponding author.

## Abbreviations

The following abbreviations are used in this manuscript:

BDHQ	Brief Self-Administered Diet History Questionnaire
BMI	Body mass index
BW	Body weight
ID	Iron deficiency
IDA	Iron deficiency anemia
IDMH	Iron deficiency with microcytosis and hypochromia
NAID	Non-anemic iron deficiency
NIDA	Non-iron deficiency anemia without reduced serum ferritin
RBC	Red blood cell count
UIBC	Unsaturated iron-binding capacity
